# Determinants of coronavirus disease 2019 vaccine acceptance, hesitancy, and barriers among healthcare workers in Ismailia, Egypt: a mixed methods study

**DOI:** 10.1186/s42506-022-00122-4

**Published:** 2022-12-12

**Authors:** Amani Waheed, Noha M. Abu Bakr Elsaid, Mayada Ghweeba, Nermine Elmaraghy, Shimaa A. Al-Touny, Nader Nemr, Rania M. Kishk, Hebatalla M. Aly

**Affiliations:** 1 Department of Public Health, Community, Occupational and Environmental Medicine, Faculty of Medicine, Suez Canal University, Ismailia, Egypt; 2 Department of Basic Medical Sciences, Faculty of Medicine, King Salman International University, South Sinai, Egypt; 3 Department of Medical Microbiology and Immunology, Faculty of Medicine, Suez Canal University, Ismailia, Egypt; 4 Department of Anaesthesia and ICU, Faculty of Medicine, Suez Canal University, Ismailia, Egypt; 5 Department of Endemic and Infectious Diseases, Faculty of Medicine, Suez Canal University, Ismailia, Egypt

**Keywords:** Coronavirus disease 19 vaccination, Vaccine hesitancy, Healthcare workers, Qualitative study

## Abstract

**Background:**

Coronavirus disease 2019 vaccine acceptance among healthcare workers (HCWs) plays a fundamental role in combating the COVID-19 pandemic. Vaccination acceptance depends on perceptions of competence and motives of the providers, producers, health professionals, and policymakers. This study aimed to identify the frequency and determinants of COVID-19 vaccine uptake acceptance, hesitancy, and barriers among HCWs.

**Methods:**

A total of 500 HCWs from 3 different hospital affiliations in Ismailia Governorate, Egypt, were included in this mixed methods study. The study was conducted between March and April 2021 through two phases. Phase 1 included a cross-sectional study using a self-administered questionnaire with inquiries about different determinants of vaccine acceptance, hesitancy, and barriers, which was completed either electronically in a Google form or a hard printed copy. Phase 2 included four focus group discussions to explore the determinants in depth.

**Results:**

The mean age of participants was 33.9 ± 7.9 years, 70% were females, 51.2% were nurses, and 28.6% were physicians. Of the 500 HCWs, only 27.8% accepted taking the vaccine immediately, 49.2% refused, and 23% were hesitant. Vaccine safety was the highest associated factor with vaccine acceptance (odds ratio (OR) = 6.3, 95% confidence interval (CI) 3.132–12.619), followed by previous uptake of influenza vaccine (OR = 3.3, 95% CI 2.048–5.217) and vaccine effectiveness (OR = 2.6, 95% CI 1.282–5.262). The main barriers to vaccine acceptance were mistrust in governmental policies during the pandemic or in the healthcare facility they work in. Hesitancy was common among females and nurses and was attributed to three prominent factors, including safety concerns, negative stories, and personal knowledge.

**Conclusions:**

The vaccine acceptance frequency among HCWs was considered low, as the majority either refused or was hesitant about taking the vaccine. Concerns about vaccine safety and effectiveness were significant determinants of vaccine acceptance. Factors related to trust were the main barriers to vaccine uptake. The health authority should establish a surveillance system for side effects of the COVID-19 vaccine and communicate this information between HCWs to decrease their worries about safety and increase vaccine uptake.

## Introduction

Since the declaration of the global coronavirus disease 19 (COVID-19) pandemic on March 11, 2020, global effort for COVID-19 vaccine research has started worldwide [1]. The economic and psychological consequences of the COVID-19 outbreak have intensified international efforts to develop a safe and effective COVID-19 vaccine, as vaccination is considered the most cost-effective method for preventing and controlling the pandemic [2]. The enforcement of quarantine and lockdown, social distancing measures, community use of personal protective measures, and travel restrictions were effective in controlling the spread of the COVID-19 infection [3]. However, it will be challenging to limit the spread of COVID-19 infection in the absence of vaccines or effective treatment [4].

As of April 8, 2022, the World Health Organization (WHO) reported 494,587,638 confirmed cases of COVID-19, 6,170,283 deaths worldwide, and 11,250,782,214 vaccine doses administered. In Egypt, there have been 276,756 confirmed cases of COVID-19 and 15,829 deaths from January 3, 2020, to June 21, 2021, as reported by WHO. As of June 20, 2021, a total of 4,010,467 vaccine doses have been administered [5]. Although the data showed excellent progress in the number of COVID-19 vaccine doses administered, there are still great challenges for upcoming COVID-19 immunization, one of which is the hesitation regarding the public acceptance of COVID-19 vaccination [6, 7]. Meanwhile, the actual vaccination rate is supposed to be much lower than the acceptance after the introduction of the vaccine [8, 9].

Since the availability of vaccines against COVID-19 for use and emergency approval by WHO, healthcare workers (HCWs) were placed first in line to receive the COVID-19 vaccines [10]. Vaccine hesitancy is defined as “a delay in acceptance or refusal of vaccination despite the availability of vaccination services” [11]. Vaccine acceptance and hesitancy are complex in nature and context-specific, varying across time, place, and perceived behavioral nature of the community, which can be affected by the excess of conflicting information and exaggerated media attention [12]. COVID-19 vaccine acceptance among HCWs plays a fundamental role in combating the COVID-19 pandemic [13]. A previous review showed that vaccination acceptance depends on perceptions of competence and motives of the providers, producers, health professionals, and policymakers [14].

HCWs have a powerful influence on their patients, families, and communities, which leads to fewer COVID-19 illnesses, hospitalizations, and deaths. However, being first in line makes some HCWs hesitant about taking the vaccine [15]. Previous studies reported that the greater hesitancy about vaccination among HCWs is associated with the lower level of knowledge about the vaccine [16, 17]. The public’s willingness to accept or decline previous pandemic vaccines showed several determinants, including the public’s perceived risk, event severity, personal consequences, and previous vaccination history [17]. Many determinants can influence vaccine decision-making, including contextual factors such as cultural, social, political, and provided vaccine information about the perceived safety and effectiveness of the vaccine [18]. Psychological traits are also considered predictors of vaccine acceptance, including perceived susceptibility to infection, benefits of vaccination, fewer barriers to vaccination, and low self-efficacy in preventing infection [19].

Understanding the determinants and confidence level of COVID-19 vaccination among HCWs is an essential element for understanding vaccine hesitancy. HCWs' acceptance of new vaccines is crucial for their protection. It is also a predictor for the community to improve vaccine uptake during pandemics [17]. Loss of COVID-19 vaccine acceptance among HCWs can lead to vaccine reluctance and refusal of the population, which results in a massive obstacle to the control of the pandemic [20]. Because HCWs are an important source of trust for health advice and vaccination uptake in high- and low-income countries, understanding key determinants that influence the preferences and demands of COVID-19 immunization by HCWs may help develop strategies for improving the national acceptance of vaccination. Therefore, this study aimed to assess the rate of COVID-19 vaccine acceptance among Egyptian HCWs and explore the determinants of vaccine acceptance, hesitancy, and barriers to vaccine uptake.

## Methods

### Study design, setting, and population

This study was conducted through a mixed methods approach using a sequential explanatory design to study COVID-19 vaccine determinants and barriers to its uptake among HCWs.

### Phase 1

A cross-sectional analytic study was conducted using a structured online/printed questionnaire to measure the COVID-19 vaccine acceptance rate and identify factors influencing COVID-19 vaccine acceptance among the study participants and hesitancy and barriers to COVID-19 vaccine uptake. In this phase, participants were recruited from three major hospitals in Ismailia: Suez Canal University Hospitals, Ministry of Health-affiliated hospitals, Suez Canal Authority-affiliated hospitals.

This study was conducted in Ismailia, which is a city in northeastern Egypt on the west bank of the Suez Canal. Ismailia City provides healthcare services to Northern Sinai, South Sinai, and Al Sharqiyah Governorates beside the Suez Canal Region. It provides its healthcare services through three major hospitals and a network of primary healthcare (PHC) centers that provide different levels of healthcare for the citizens in these areas. They all benefit from the services of the PHC facilities, including COVID vaccination.

The study population included physicians, pharmacists, nurses, and health technicians working in these hospitals.

### Sampling

#### Sample size [21]

$$n={\left[\frac{{\textrm{Z}}_{\propto /2}}{\textrm{E}}\right]}^2\ast \textrm{P}\left(1-\textrm{P}\right)$$

where *n* indicates the sample size, Z α/2 = 1.96 (the critical value that divides the central 95% of the *Z* distribution from the 5% in the tail), *P* indicates the prevalence of the acceptance rate among HCWs = 36% [12], and *E* indicates the margin of error (width of confidence interval (CI)) = 5%. So, the estimated sample size was 354.

To perform logistic regression analyses, the sample was increased to a minimum of 500 to derive statistics representing the parameters [22].

##### Sampling technique

A list of all HCWs was obtained from each participating hospital, and then these lists were merged into one complete list. The required sample was selected by a simple random method from the list, including physicians, pharmacists, nurses, and technicians.

The names of the selected participants were sent to the IT Department in each hospital to send them the Google form on their e-mails or WhatsApp accounts or to give them a hard printed copy of the questionnaire to fill.

The Google form link included informed consent about the study and authors at the beginning where participants have to accept or deny participation in the study before proceeding to the questions, and participants must provide consent before proceeding with the questionnaire. In the Google form, the options of “collect e-mail addresses” and “limit to one response” were activated to avoid multiple responses by the same subject.

For participants who filled in a hard copy, they must add their e-mail addresses at the beginning of the questionnaire and provide informed consent.

### Data collection tool

Data were collected from March 1 to April 1, 2021, using a self-administered questionnaire. We reviewed the literature and extracted the study tool from the previously used validated questionnaires [16, 23]. It was planned to be easy, short, and clear for the participants. The questionnaire’s content and clarity were assessed by public health experts at Suez Canal University with no modification. The questionnaire consisted of 45 items divided into the following sections:

1. Sociodemographic characteristics, including age, gender, marital status, and education.
2. Perceived perception of risk and severity of COVID-19, including the probability of getting infected, previous exposure to MERS-CoV- or COVID-19-infected patients, whether HCWs themselves were ever infected with COVID-19, and worry about spreading infection among family members and death of any family member or friends due to COVID-19 infection.
3. COVID-19 vaccine knowledge, including knowing the availability of vaccines for HCWs in the hospitals, sources of information regarding the vaccine, and knowing the different types of vaccines.
4. COVID-19 vaccine acceptance status (strongly agree, agree, do not know, disagree, and strongly disagree).
5. Hesitancy and barriers to vaccine uptake, including insufficient evidence, concern about vaccine side effects, lack of confidence about vaccine effectiveness and safety, mistrust in governmental policy during the pandemic, and mistrust in health information system. The online questionnaire was distributed and available at the following link: https://docs.google.com/forms/d/1gQ%2D%2DwEWsTREa96YyH39j80FC3SNmE4F5n7_enWXw8yY/edit?usp=sharing.

### Phase 2

A qualitative study was conducted through focus group discussions (FGDs) to explore the determinants of COVID-19 vaccine acceptance, barriers, and attitudes among HCWs. Participants were purposively selected from HCWs of the selected hospitals. The sampling was guided by a sampling framework designed to recruit physicians and nurses with various medical specialties. A total of 23 HCWs participated in this qualitative study (12 nurses and technicians and 11 physicians from different medical specialties). We conducted four FGD sessions, with an average duration of 69 min (range 56–82 min).

FGDs were conducted either at the Suez Canal University Hospitals or through telecommunications applications (Zoom meetings). All the interviews were audio recorded. Written consent was obtained from all participants of the qualitative study at the start of each FGD. Participants of the online interviews sent a photo of their signed consent form after reading the information sheet of the study. Participants were asked to complete a brief questionnaire before the beginning FGDs. This questionnaire included demographic information (age, gender, marital status, occupation, and their position at the hospital). A semi-structured interview questionnaire with open-ended questions was used to allow the participants to explain their views and thoughts. The interview guide included questions on personal attitudes regarding COVID-19 vaccine acceptance, common health information sources sought by participants, the effectiveness of personal protective equipment compared to vaccination, and motivators for COVID-19 as a family or social pressure for vaccination. Probes were used when FGDs came to an end or deviated from the study topic.

All the FGD sessions were conducted in Arabic. All recorded data were translated into English and transcribed manually by the interviewers. Data collection continued until saturation was reached or when no new emerging themes were noted [24]. A thematic analysis method was used to analyze the recorded data. All the interview transcripts were analyzed independently by the main interviewers. Data were categorized into codes, themes, and subthemes. Inductive analysis was performed, but existing theoretical frameworks were not used.

### Statistical analysis

For quantitative data, descriptive statistical methods were used to summarize data on sociodemographic characteristics and association with variables. Comparison between participants who accepted taking the vaccine immediately, those who were hesitant, and those who did not accept taking the vaccine regarding different determinants was performed using chi-square and Fisher’s exact tests. Binary logistic regression analysis was performed to identify factors associated with vaccine acceptance among the participants.

All data analyses were performed using Statistical Package for the Social Sciences software, version 23. A *p* value of < 0.05 was considered statistically significant.

Qualitative data were translated, transcribed, and analyzed manually using the thematic analysis approach. The main data sources were the transcribed audio-recorded interviews, the notes summary collected in each discussion, and the questionnaire form used at the beginning of the discussion. The categories used for the framework were informed by our research questions and sensitive to topics emerging from the data analysis. After the codes were extracted, we explored the framework categories for relationships. The initial codes were then classified into themes and subthemes. These themes were reevaluated to ensure that each theme had sufficient supporting data.

### Ethical considerations

The study was approved by the Research Ethics Committee of the Faculty of Medicine, Suez Canal University, on February 22, 2021 (reference 4478). Informed consent was obtained from all participants before participation. The collected data were kept confidential for only research use. Feedback on the study results was announced to the participants at the end of the study and to the work site managers. In phase two, all participants provided consent before conducting the FGDs. All sessions were audio recorded with the participants’ consent, and anonymity was assured. Participants had the option of choosing a nickname to be used during interviews and data analysis.

## Results

### Phase 1 of the study

A total of 500 HCWs were enrolled in this study. The mean age of participants was 33.9 ± 7.9 years (range 20–73 years). About 70% were females. Among the female participants, 5.7% were pregnant. About 72% were married, 23% were single, and the remaining were divorced or widows. Furthermore, 45% had secondary school education, 21.4% had bachelor’s degrees, and 33.6% had postgraduate degrees.

Regarding occupation, more than half of the participants (51.2%) were nurses, 28.6% were physicians, 12.4% were pharmacists, and 7.8% were technicians. The highest percentage (57.2%) were working in Suez Canal University Hospitals, 24.8% were working in Ministry of Health-affiliated hospitals, and 18% were working in Suez Canal Authority-affiliated hospitals.

**Fig. 1:**
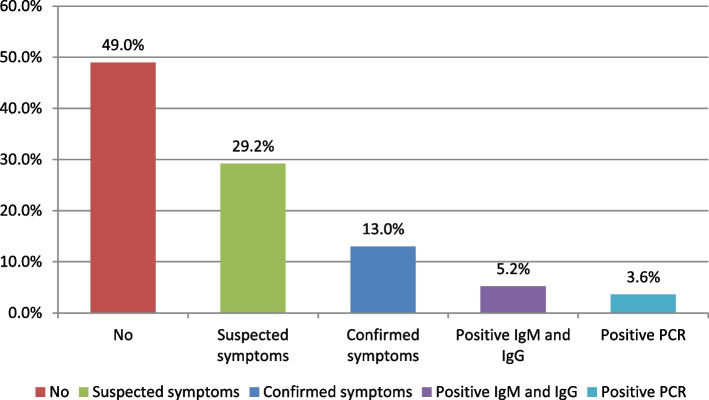
COVID-19 infection history among participating healthcare workers from Ismailia, Egypt in the 1st half of 2021 (*n* = 500)

The participants’ history of COVID-19 infection was presented as a confirmed infection by polymerase chain reaction (PCR) or positive immunoglobulin M or immunoglobulin G test, confirmed symptoms or suspected symptoms, or never having an infection (Fig. 1). Additionally, 49% were not infected with COVID-19 before.

**Fig. 2:**
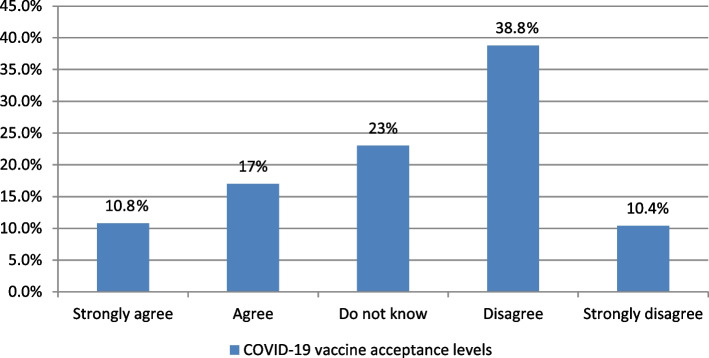
Vaccine acceptance levels among the participating healthcare workers from Ismailia, Egypt in the 1st half of 2021 (*n* = 500)

Participants were asked about their acceptance of vaccine uptake immediately, and their answers were ranked on a Likert scale (strongly agree, agree, do not know, disagree, and strongly disagree) (Fig. 2). Responses of strongly agree or agree were assigned as accepting (about 28% of participants), responses of strongly disagree or disagree were assigned as not accepting (49% of participants), and responses of do not know were assigned as hesitating (about 23% of participants).

Table 1 shows the differences between the three groups regarding the different determinants. Males, those having children, physicians, and those working in Suez Canal Authority-affiliated hospitals showed higher frequencies of vaccine acceptance than others.

**Table 1:** Determinants and barriers of COVID-19 vaccine acceptance among healthcare workers in the studied hospitals according to the state of acceptance and hesitancy to take the vaccine during March and April 2021, Ismailia, Egypt

Determinants	Not accepting (***n*** = 246) Frequency (%)	Accepting (***n*** = 139) Frequency (%)	Hesitating (***n*** = 115) Frequency (%)	***p*** value
**Socio-demographic factors**				
Gender				
• Male	59 (38.8)	65 (42.8)	28 (18.4)	**< 0.001**^1*^
• Female	187 (53.7)	74 (21.3	87 (25)	
Education level				
• Middle education	129 (57.3)	44 (19.6)	52 (23.1)	
• Higher education	57 (53.3)	32 (29.9)	18 (16.8)	**< 0.001**^1*^
• Postgraduate education	60 (35.7)	63 (37.5)	45 (26.8)	
Occupation				
• Physician	53 (37.1)	57 (39.9)	33 (23.1)	
• Pharmacist	29 (46.8)	15 (24.2)	18 (29)	**0.002**^2*^
• Nurse	144 (56.3)	54 (21.1)	58 (22.7)	
• Technician	20 (51.3)	13 (33.3)	6 (15.4)	
Place of work				
• SCU teaching hospital	156 (54.5)	59 (20.6)	71 (24.8)	
• MoH affiliated hospital	59 (47.6)	41 (33.1)	24 (19.4)	**< 0.001**^1*^
• SCA hospital	31 (34.4)	39 (43.3)	20 (22.2)	
Having children				
• No	59 (59)	27 (27)	14 (14)	**0.023**^1*^
• Yes	163 (46)	99 (28)	92 (26)	
**COVID-19 history and perception of infection risk**				
Anxious about infection				
• No	69 (60)	31 (27)	15 (13)	**0.007**^1*^
• Yes	177 (46)	108 (28.1)	100 (26)	
**SARS-CoV 2 virus and pandemic perception**				
Do you think that SARS CoV 2 is a natural virus that spread accidentally?				
• No	160 (55.0)	66 (22.7)	65 (22.3)	**0.003**^1*^
• Yes	86 (41.1)	73 (34.9)	50 (23.9)	
**Knowledge and perception about COVID-19 vaccine**				
Has your information about the vaccine helped you to make a decision regarding accepting or refusing to uptake the vaccine?				
• No	118 (56.5)	33 (15.8)	58 (27.8)	**< 0.001**^1*^
• Yes	128 (44)	106 (36.4)	57 (19.6)	
Do you think that the vaccine had a role in stopping the pandemic?				
• No	183 (67.5)	29 (10.7)	59 (21.8)	**< 0.001**^1*^
• Yes	63 (27.5)	110 (48)	56 (24.5)	
Do you think that the vaccine had a role in reducing disease complications?				
• No	154 (69.1)	16 (7.2)	53 (23.8)	**< 0.001**^1*^
• Yes	92 (33.2)	123 (44.4)	62 (22.4)	
Do you think that the vaccine is effective?				
• No	193 (68.4)	23 (8.2)	66 (23.4)	**< 0.001**^1*^
• Yes	53 (24.3)	116 (53.2)	49 (22.5)	
Do you think that the vaccine is safe?				
• No	229 (60.7)	43 (11.4)	105 (27.9)	**< 0.001**^1*^
• Yes	17 (13.8)	96 (78)	10 (8.1)	
Do you think that evidence regarding vaccine safety and effectiveness is sufficient?				
• No	229 (53.4)	91 (21.2)	109 (25.4)	**< 0.001**^1*^
• Yes	17 (23.9)	48 (67.6)	6 (8.5)	
Do you think that the vaccine has serious side effects?				
• No	75 (40.8)	81 (44)	28 (15.2)	**< 0.001**^1*^
• Yes	171 (54.1)	58 (18.4)	87 (27.5)	
Do you think that the vaccine can infect you with the virus?				
• No	75 (38.7)	83 (42.8)	36 (18.6)	**< 0.001**^1*^
• Yes	171 (55.9)	56 (18.3)	79 (25.8)	
Are you vaccine opponent in general?				
• No	158 (40.7)	132 (34)	98 (25.3)	**< 0.001**^1*^
• Yes	88 (78.6)	7 (6.3)	17 (15.2)	
**Trust in governmental policies in dealing with the pandemic and trust in healthcare facility**				
Do you trust governmental policy in dealing with the pandemic?				
• No	145 (57.1)	50 (19.7)	59 (23.2)	**< 0.001**^1*^
• Yes	101 (41.1)	89 (36.2)	56 (22.8)	
Do you trust the healthcare facility that you work for?				
• No	127 (57.7)	38 (17.3)	55 (25)	**< 0.001**^1*^
• Yes	119 (42.5)	101 (36.1)	60 (21.4)	
Do you accept the use of emergency law to vaccinate the public?				
• No	84 (61.8)	26 (19.1)	26 (19.1)	
• Yes	13 (18.6)	44 (62.9)	13 (18.6)	**< 0.001**^1*^
• Do not know	149 (50.7)	69 (23.5)	76 (25.9)	
Do you think medical mistakes are common?				
• No	14 (32.6)	18 (41.9)	11 (25.6)	
• Yes	164 (48.4)	101 (29.8)	74 (21.8)	**0.010**^1*^
• Do not know	68 (57.6)	20 (16.9)	30 (25.4)	
**History of influenza vaccine**				
Have you received seasonal influenza vaccine previously?				
• No	193 (55.6)	66 (18.9)	88 (25.4)	**< 0.001**^1*^
• Yes	53 (34.6)	73 (47.7)	27 (17.6)	
How many times did you receive it in the past 3 years?				
• Once	36 (40.4)	37 (41.6)	16 (18)	
• Twice	13 (29.5)	23 (52.3)	8 (18.2)	
• 3 times	8 (40.0)	5 (25.0)	7 (35)	**< 0.001**^1*^
• Never	189 (54.5)	74 (21.3)	84 (24.2)	

*SCU* Suez Canal University, *MoH* Ministry of Health and population

Data are presented as frequency and percentage;

^1^Chi square test

*Statistically significant at *p* < 0.05

The most important determinants of vaccine hesitancy were female gender, being a nurse, secondary education level, and working in Suez Canal University Hospitals. The higher hesitancy rate was among those who thought that there was no sufficient evidence regarding vaccine safety and effectiveness (94.7%), the vaccine was not safe (91.3%), and the vaccine had serious side effects (75.6%).

Trust in governmental policies during the pandemic and trust in the healthcare facility they work in were the most frequent barriers to vaccine acceptance. More than two-thirds (67.8%) of the participants thought that medical mistakes are common, 8.6% thought that they are not, and 23.6% did not know. This reflects participants’ trust in health professionals, which is another barrier to vaccine acceptance.

Only 14% accepted the use of emergency law to vaccinate the public, and the remaining either refused or did not know.

Furthermore, two logistic regression models were used to determine the best predictor of vaccine acceptance among the participating HCWs. The first model included sociodemographic factors, previous intake of influenza vaccine, and trust in the healthcare facility they work in. The other model included knowledge and beliefs regarding the COVID-19 vaccine. Table 2 shows the two models. From the second model, vaccine safety was the best predictor with an odds ratio (OR) of 6.28 (95% CI 3.132–12.619, *p* < 0.001), followed by vaccine effectiveness (OR = 2.6, 95% CI 1.282–5.262, *p* = 0.008) and sufficient evidence regarding vaccine (OR = 2.5, 95% CI 1.154–5.508, *p* = 0.020). From the first model, the best predictor was the previous uptake of influenza vaccine (OR = 3.3, 95% CI 2.048–5.217, *p* < 0.001), followed by education level (OR 1.5, 95% CI 1.123–1.921, *p* = 0.005).

**Table 2:** Regression models for determinants of COVID-19 vaccine acceptance among HCWs recruited from the participating hospitals in Ismailia, Egypt, during March and April 2021

	β	***p*** value	OR (95% CI)
**Model 1**			
Gender (male/female)	− 0.657	0.007^*^	0.518 (0.321–0.837)
Education	0.384	0.005^*^	1.469 (1.123–1.921)
Having children (0/1)	− 0.040	0.888	0.961 (0.555–1.665)
Do you think SARS-CoV-2 is natural? (0/1)	0.402	0.087	1.495 (0.943–2.370)
Have you ever taken influenza vaccine previously? (0/1)	1.184	< 0.001^*^	3.268 (2.048–5.217)
Anxious about infection (0/1)	0.101	0.772	1.106 (0.635–1.925)
Do you trust the healthcare facility that you work in? (0/1)	0.527	0.087	1.692 (0.927–3.087)
Constant	− 1.895	0.004^*^	0.150
Model ϰ^2^ = 82.669		< 0.001^*^	
**Model 2**			
Has your information regarding COVID-19 vaccines helped you to make your decision? (0/1)	0.680	0.025^*^	1.973 (1.088–3.579)
Do you think that the vaccine plays a role in stopping the pandemic? (0/1)	0.672	0.048^*^	1.959 (1.007–3.810)
Do you think that the vaccine plays a role in reducing disease complications? (0/1)	0.271	0.492	1.311 (0.605–2.839)
Do you think that the vaccine has serious side effects? (0/1)	− 0.226	0.478	0.798 (0.428–1.489)
Do you think that the vaccine is effective? (0/1)	0.954	0.008^*^	2.597 (1.282–5.262)
Do you think that the vaccine is safe? (0/1)	1.838	< 0.001^*^	6.286 (3.132–12.619)
Do you think that the evidence regarding vaccine safety and effectiveness is sufficient? (0/1)	0.925	0.020^*^	2.521 (1.154–5.508)
Do you think that the vaccine can infect you with the virus? (0/1)	− 0.473	0.623	0.623 (0.339–1.143)
Are you a vaccine opponent in general? (0/1)	− 1.717	0.180	0.180 (0.062–0.517)
Constant	− 2.649	0.071	0.071
Model ϰ^2^ = 252.579		< 0.001^*^	

The outcome variable is vaccine acceptance versus not accepting/hesitating. *Statistically significant at *p < 0.05*

### Phase 2 of the study

The analysis of the qualitative study showed a mixed view regarding the approach toward the COVID-19 vaccine. The findings were classified into two main themes: (1) impact of the available health information about COVID-19 vaccines, which included subthemes: confusion, distress, and mistrust, and (2) vaccine hesitancy and acceptance. The subthemes of confusion, distress, and mistrust were interconnected and overlapped. Generally, the more the confusion, distress, and mistrust felt by participants about the vaccine, the less likely they were willing to accept vaccination for themselves or for their family members.

#### Information and misinformation about the COVID-19 vaccine

Participants mentioned various sources of COVID-19 health-related information, such as TV and radio, the Internet including Google, social media such as YouTube and Facebook, and medical journals. Participants expressed mistrust in some traditional sources of news, which was more obvious for those who search for COVID-19-related news in other countries’ news or international scientific journals. This affected how people viewed their COVID-19 vaccination decision. Many participants decided to avoid watching the news about COVID-19, particularly when faced with contradictory information. A 42-year-old female nurse noted:

“Whenever I open my phone, I found a lot of information about COVID-19. This information comes from everyone not only from doctors or the Ministry of Health, but it comes from everybody. This makes me more worried about myself and my family.”

A 27-year-old physician noted, “From all this mess, I decided to ignore it all. I don’t read any health information about Corona from any source. Sometimes I do not believe what was said by the Ministry of Health.”

Misinformation about the vaccine, particularly passive information, has the potential to affect participants’ vaccination decisions. For instance, a female participant mentioned that she was keen to take the vaccine, and she changed her mind after listening to a story about people who were injected with the COVID-19 vaccine and developed serious side effects.

All participants expressed a desire to follow the hospitals’ rules and restrictions during the pandemic. However, they felt that the guidelines for HCWs' vaccination were confusing. Participants explained that the provided health information about the vaccination was poor, and the decisions contradicted their hospital’s policy for the availability of COVID-19 PCR testing. A 48-year-old female nurse noted:

“It is not understandable when you provide me with a free vaccine and at the same time you ask me to pay 1000 pounds for the test.”

In this theme, participants listed a range of misinformation they had encountered regarding COVID-19, resulting in confusion, distress, and mistrust. Participants agreed that providing them with trustworthy health information resources by the Suez Canal University Hospitals will promote their attitude toward COVID-19 vaccination acceptance.

#### Vaccine acceptance and hesitancy

This study showed a high level of vaccine hesitance among HCWs, particularly nurses. Of the 23 HCWs interviewed in this study, only 2 participants had their first dose of the vaccine, five had a mixed view with concerns about COVID-19 vaccine safety, and the remaining participants were unwilling to accept the COVID-19 vaccine.

The safety of the COVID-19 vaccine was a major concern for all participants. They questioned how quickly the vaccine had been produced and whether the side effects had enough time to be fully tested. These worries had been exacerbated by engagement with social media stories. A 29-year-old nurse preferred to wait for at least 6 months to test the effect of the vaccine on others before taking it. She noted:

“I’m not convinced yet that it is a good idea to take the vaccine. And if I take it, I will wait at least six months to see if there are any side effects happened to those who took it.”

Some concerns about the future effects of the vaccine on comorbid and immunocompromised patients were mentioned by many participants. A 31-year-old female nurse with a thyroid health problem noted:

“For me, I may think of taking the vaccine. But not now I want to be sure that there will be no future side effects that could happen to me.”

Participants, mostly physicians, agreed that the manufacturing of the COVID-19 vaccine is a fundamental method to control the pandemic. Therefore, they agreed to take any vaccine provided in the Suez Canal University Hospitals. The idea that the acceptability of the COVID-19 vaccine among physicians will impact the overall acceptability in the general population was agreed upon by all participants. Few participants showed confidence in any vaccine provided. They believed that when providing a vaccine in the market, people should have some confidence that it was a good vaccine and that it was quite safe.

A 26-year-old participant noted, “I believe they would never put the unsafe vaccine in the market, you know it is a great responsibility, and if you think it is not effective, it will not cause any harm.”

Vaccine hesitancy could be attributed to three prominent factors: safety concerns, negative stories, and personal knowledge. Vaccine acceptance was higher among physicians than among nurses. Vaccine safety and potential comorbidity side effects were the main concerns about vaccine acceptance among all participants.

## Discussion

To our knowledge, this study is one of the initial studies to assess determinants of vaccine acceptance, hesitancy, and barriers to vaccine uptake in Egypt using a mixed methods study. In this study, only 28% of participants accepted taking the COVID-19 vaccination immediately when available, which is considered low. Most of the participants were hesitating or refused to take the vaccination. This could be due to the time of conduction of our study, as we assessed acceptance a few months after the initiation of vaccination in Egypt. These results were consistent with those reported in an early survey between October 7 and November 9, 2020, in the USA, showing that only 36% of HCWs were willing to take the vaccine immediately, and more than half of the HCWs chose to postpone their decision until evaluating more statistics [13]. Furthermore, this was consistent with a study on HCWs in Saudi Arabia that reported a frequency of 33% vaccine acceptance immediately after 1 month of the vaccine administration [25]. In contrast, high acceptance rate of vaccination among HCWs was found in Bangladesh (85%) [26], Pakistan (70%) [27], Canada (80.9%) [28], and the UK (64%) [29]. Furthermore, a higher rate of acceptance was reported in Kuwait (83.3%) [30] and Saudi Arabia (64.7%) [15]. The variation in the rates of acceptance between countries and even within the same country, as in Saudi Arabia, could be explained by the effect of time. When new information becomes available, policies change, and new vaccines appear. Moreover, studying the intention to take the vaccine differs from studying the actual intake of the vaccine.

In this study, sociodemographic factors, such as male gender and higher education levels, showed higher vaccine acceptance frequencies than other categories. However, age and marital status did not show statistically significant differences in vaccine acceptance. In agreement with these findings, a study in China showed that the male gender was a positive predictor for the acceptance of the COVID-19 vaccine [8]. This result could be due to the reported high rates of COVID-19-related morbidity and mortality among male COVID-19 patients. Similarly, another study in Turkey showed that the acceptance rates were higher in men [31]. Contrary to our study, COVID-19 vaccine acceptance was found to increase with the increase of age [28].

In this study, the highest percentage of vaccine acceptance was among physicians (39.9%), followed by technicians (33.3%) and nurses (21.1%), and this difference was statistically significant. This may be due to the diversity of the sources of information regarding vaccines and the differences in their roles in patients’ care. This finding was consistent with a previous study in the USA, which reported that vaccine acceptance varied among different occupational classes of HCWs, and physicians had higher vaccine acceptance, followed by administrative staff and nurses [13]. Similarly, Al-Sanafi and Sallam concluded that physicians had higher frequencies of COVID-19 vaccine acceptance than nurses [30].

The results of this study showed that being anxious about the risk of COVID-19 infection increased the rate of vaccine acceptance. Previous studies reported the perceived risk of becoming infected as a predictor behind the intention for vaccination [30, 32].

Furthermore, a significant relationship was observed between participants’ decisions on vaccination and knowledge, attitude, and beliefs toward COVID-19 vaccination. Most of the participants who accepted vaccination thought that the vaccine is effective and safe and plays a role in stopping the pandemic and reducing complications. Participants who thought that the vaccine is safe were 6.3 times more likely to accept the vaccine than those who did not think that it is safe. In agreement with our study, Shekhar et al. reported that some concerns specific to COVID-19 vaccination among HCWs who do not plan to take the COVID-19 vaccine were prevalent regarding the vaccine efficacy, adverse effects, and rapidity of development [13].

The results showed that the main barriers to vaccine acceptance were related to HCWs’ trust in the healthcare facilities they work in. Those HCWs were 2.7 times more likely to accept the vaccine than those who did not trust the healthcare facility they work in. Another barrier was the lack of trust in health professionals. About 68% of our participants thought that medical mistakes were common. This might indicate an important role for the propagation of information through medical organizations and professional societies to increase the uptake among HCWs. Meanwhile, a recent study reported poor trust in regulatory authorities and government among HCWs who did not want to be vaccinated with high trust in their medical professionals prescribing the vaccine [13]. Also, similar to our findings, a previous study among HCWs in Canada found that refusal was more probable when users had a lack of trust in health experts and in pharmaceutical companies who provide the vaccine [28]. Additionally, previous studies have found that participants with higher levels of confidence in the healthcare system are more likely to accept the vaccine [16, 33]. A recent scoping review also showed that mistrust in authorities, health experts, and pharmaceutical companies was associated with vaccine hesitancy [34].

This study revealed a significant relationship between receiving the influenza vaccine and accepting the COVID-19 vaccine; that is, participants who agreed to take the vaccine had previously taken the seasonal influenza vaccine. This is confirmed by the results of a scooping review by Biswas et al., who concluded that history of influenza vaccine increases COVID-19 vaccine acceptance, and this was shown in more than half of the studies in the review [34]. This may reflect that vaccine acceptance may be related to the vaccination behavior of participants.

In this study, 23% of HCWs were hesitating and had not yet decided to take the COVID-19 vaccination. This was in parallel with the findings of the review conducted by Biswas et al. in 2021, who reported that 22.5% of HCWs worldwide showed hesitancy about the COVID-19 vaccine [34]. The frequency of vaccine hesitancy is also consistent with a recent study conducted in France, Belgium, and Quebec, which reported that 23% of HCWs were hesitant about taking the vaccine [35].

This study found that COVID-19 vaccine hesitancy could be attributed to three prominent factors: safety concerns, negative stories, and personal knowledge. A higher rate of hesitancy was among those who thought that the vaccine was not safe or can cause serious side effects. Additionally, COVID-19 vaccine hesitancy was higher among participants with chronic health conditions. These findings were consistent with a study conducted in India, which concluded that trust in the perceived safety of vaccines and vaccine side effects is a prime contributing factor to public willingness to accept the COVID-19 vaccine [36]. Worries regarding a lack of long-term studies, concerns about the vaccine’s efficiency, and adverse effects were some of the most common hurdles raised in previous studies [37, 38]. Barriers driving participants’ unwillingness to obtain immunization were also reported in a previous study in Iraq [11].

This study showed that vaccine safety was the main driver for vaccination decision-making. This was consistent with a previous study, which showed that this factor was a key determinant, especially for newly introduced vaccines that have not been fully tested in the real world [39]. Similarly, providing reliable health information through consistent national efforts is of paramount importance for tackling COVID-19 vaccine hesitancy among HCWs, which is consistent with previous studies [40, 41]. Building trust in vaccine safety and efficacy is crucial for increasing immunization coverage, despite the fact that it will require a great effort [42, 43].

The low acceptance rate of the COVID-19 vaccine in this study could also be attributed to another factor, which is the timing of data collection. Data were collected in March 2021, just after the initiation of the vaccine in Egypt and before the peak of the third wave, and in that period data regarding COVID-19 vaccine effectiveness and safety and surveillance of post-COVID-19 vaccination symptoms were scarce and not readily available.

### Study strengths and limitations

The main strength of this study is that it was conducted using a mixed methods study, which increases the understanding of the results. The study included different occupational categories (physicians, pharmacists, nurses, and technicians), and participants were recruited by a simple random method; this gave better representativeness of HCWs from different facilities with different affiliations. As few studies have investigated the COVID-19 actual vaccine uptake acceptance and hesitancy, especially among HCWs, this study will serve as a source of data that could be useful for estimating the trends of vaccine acceptance levels among HCWs in our country and the Arab world. This was a cross-sectional study, so we could not determine the predictive role of each associated variable that should be calculated from prospective studies. The study provides only a snapshot in time and does not yield incidence. Finally, the study findings were based on self-reported information, which may be subjected to information bias.

## Conclusions

The vaccine acceptance rate among HCWs at the time of conducting the study was considered low. Only 28% of HCWs accepted taking the vaccine, 49% did not accept, and 23% were hesitating and had not decided yet. Male sex and higher education levels were significant determinants of vaccine acceptance. Participants who thought that the vaccine is safe were more likely to accept the vaccine than those who did not think that it is safe. The lack of trust in governmental policies and the healthcare facility they work in was the most frequent barrier to vaccine acceptance. Determinants of vaccine hesitancy were female gender, being a nurse, secondary education level, misbeliefs and negative attitude toward vaccine safety, serious side effects, and the presence of insufficient evidence concerning vaccine safety and effectiveness.

Based on our findings, we recommend that the health authority should establish a surveillance system for side effects of the COVID-19 vaccine and communicate this information between HCWs to decrease their worries regarding safety and increase vaccine uptake. This will also build trust between the healthcare facility and HCWs, which will be reflected to the public. Health authorities should raise public confidence in COVID-19 vaccinations, considering the influence of these beliefs on vaccine hesitancy. Since the highest levels of vaccine hesitancy were observed among certain HCWs (females, nurses, and workers with secondary education), focused awareness interventions should target these groups first.

## Data Availability

The datasets used and/or analyzed in the current study are available from the corresponding author upon reasonable request.
